# Efficacy and bleeding risk of antithrombin supplementation in septic disseminated intravascular coagulation: a secondary survey

**DOI:** 10.1186/s13054-014-0497-x

**Published:** 2014-09-15

**Authors:** Toshiaki Iba, Daizoh Saitoh, Hideo Wada, Hidesaku Asakura

**Affiliations:** Department of Emergency and Disaster Medicine, Juntendo University Graduate School of Medicine, 2-1-1 Hongo, Bunkyo-ku, Tokyo 113-8421 Japan; Division of Traumatology, Research Institute, National Defense Medical College, 3-2 Namiki, Tokorozawa-shi, Saitama 359-8513 Japan; Department of Molecular and Laboratory Medicine, Mie University Graduate School of Medicine, Tsu, Mie 514-8507 Japan; Department of Internal Medicine III, Kanazawa University School of Medicine, 13-1 Takaramachi, Kanazawa, Ishikawa 920-8641 Japan

## Abstract

**Introduction:**

In a previous report, we demonstrated a favorable trend for supplementation with antithrombin (AT) concentrate at a dosage of 3,000 IU/day over 1,500 IU/day for the treatment of sepsis-associated disseminated intravascular coagulation (DIC) in patients with an AT activity of 70% or less. Since the survival difference did not reach statistical significance, we planned to examine the effects in a larger number of cases with severer disease.

**Methods:**

We performed a non-randomized multi-institutional survey. In total, 307 septic DIC patients who had AT activity less than 40% and who had undergone AT substitution at a dose of either 1,500 IU/day or 3,000 IU/day for three consecutive days were analyzed. Of these, 259 patients received 1,500 IU/day (AT1500 group) and 48 patients received 3,000 IU/day (AT3000 group). The primary efficacy endpoints were recovery from DIC by day 7 and an all-cause mortality on day 28. Adverse bleeding events were also examined. A logistic regression analysis was conducted by using age, sex, body weight, initial AT activity, DIC score, platelet count, coadministration of heparin, recombinant thrombomodulin, suspected source of infection, surgery, and supplemented AT dose.

**Results:**

Supplementation significantly decreased the DIC score in the AT3000 group, leading to the superior resolution of DIC, compared with the results in the AT1500 group (66.7% versus 45.2%, *P* = 0.007). In addition, the AT3000 group exhibited a better survival than the AT1500 group (77.1% versus 56.4%, *P* = 0.010). Bleeding events were observed in 6.96% (severe bleeding: 3.04%) in the AT1500 group and 6.52% (severe bleeding, 4.35%) in the AT3000 group (*P* = 1.000; severe bleeding, *P* = 0.648). A logistic regression analysis revealed that the use of AT3000 (odds ratio (OR), 2.419; *P* = 0.025), a higher initial platelet count (OR, 1.054; *P* = 0.027), and patient age (OR, 0.977; *P* = 0.045) were significantly correlated with an improved survival.

**Conclusions:**

The AT3000 group exhibited significantly improved rates of survival and recovery from DIC without an increased risk of bleeding, compared with the AT1500 group, among the patients with sepsis-associated DIC and an AT activity of less than 40%.

## Introduction

Severe sepsis is almost consistently associated with a hyper-coagulatory status [1] and frequently leads to disseminated intravascular coagulation (DIC) [2]. Excess clot formation, leading to malcirculation in organs and subsequent organ failure, is driven by activated coagulation, by impaired anticoagulant mechanisms, including the antithrombin (AT) and protein C system, and by the depression of the fibrinolytic system [3]. Thus, supplementation with AT concentrates has been considered a rational therapy [4].

Previously, the results of a large-scale randomized controlled trial (RCT) known as KyberSept, which examined the effects and adverse events of high-dose AT for the treatment of severe sepsis, were published [5]. This trial demonstrated that AT did not provide survival benefit and instead increased the risk of bleeding. Since AT, particularly when administered with heparin, increases the risk of bleeding significantly, the international guidelines for severe sepsis recommend “not to use high-dose AT for severe sepsis” [6]. Since the completion of KyberSept, no high-powered study has been repeated; however, a systematic review of AT use in patients with DIC with severe sepsis concluded that AT might increase the overall survival [7]. Furthermore, Fourrier [8] demonstrated an improvement in all-cause mortality across subgroups defined according to the DIC status at entry in RCTs of AT and activated protein C. Therefore, it may be too early to conclude that AT is not effective for sepsis-associated DIC.

Recently, the Japanese Association for Acute Medicine (JAAM) DIC study group performed a prospective RCT and reported that 30 IU/kg per day of AT could decrease the DIC score, compared with a placebo, in septic DIC patients with a baseline AT activity level of between 50% and 80%, which subsequently resulted in an increase in the DIC resolution rate [9]. Unlike high-dose administration, the supplementation of AT, in which the recovery of AT activity to within the normal range is targeted, has been thought to be beneficial in patients with septic DIC in Japan [10,11]. As a result, AT supplementation was included in the Japanese guidelines for sepsis-associated DIC [12]. However, a lack of sufficient evidence has also been pointed out [13]. Under these circumstances, the recent international guideline for the management of DIC has also recommended the use of AT in situations in which the efficacy of such treatment has been proven [13]. Therefore, the examination of the effects and the adverse events associated with this therapy has become an urgent matter.

In a former study, we examined a total of 729 sepsis-associated DIC patients with AT activity levels of 70% or lower and reported that 3,000 IU/day of AT substitution for 3 days (AT3000) was a significant factor associated with an improvement in survival, compared with 1,500 IU/day of AT substitution (AT1500) (odds ratio (OR) 1.912; *P* = 0.026). However, the survival difference between AT3000 and AT1500 was not statistically significant when examined by using a Fisher’s exact test [14]. The other finding in our study was that AT3000 was more effective in a patient population with a lower baseline AT activity. Thus, we further accumulated septic DIC patients with a baseline AT activity of less than 40%. The primary goal of the present study was to confirm an improvement in the resolution of DIC by day 7 and a reduction in all-cause mortality on day 28.

## Materials and methods

### Definitions

Both systemic inflammatory response syndrome (SIRS) and sepsis were defined according to the definition of the American College of Chest Physicians/Society of Critical Care Medicine consensus conference [15]. The severity of the illness of the patients was evaluated according to the AT activity [16], JAAM DIC score [17], and Sepsis-Related Organ Failure Assessment (SOFA) score at the time of enrollment. DIC was defined on the basis of the JAAM DIC criteria [18], and the DIC scores were calculated by using this system. The scoring system consists of the SIRS score, platelet count, fibrin/fibrinogen degradation product (FDP), or D-dimer and prothrombin time ratio; these values were measured in local laboratories. Organ failure was assessed by using the SOFA score [19]. Bleeding events were recorded for 28 days. Bleeding was classified as major if it was intracranial or required a transfusion of at least 3 units of blood. Less-severe bleeding was defined as minor bleeding [5].

### Patient selection and data collection

This survey was performed as a non-randomized, multi-institutional, post-marketing survey. In total, 307 sepsis-associated DIC patients with an AT activity of less than 40% who were treated in 217 hospitals between May 2006 and March 2013 were examined in this study. Patients with a history of allergic shock reaction to AT were excluded. The study was conducted in accordance with the Declaration of Helsinki and Good Post-marketing Surveillance Practice (Good Vigilance Practice and Good Post-marketing Study Practice). The institutional review boards that approved this study can be found in the Acknowledgments section. The patients’ agreement and consent were obtained when required by the ethics committee of each hospital (between May 2006 and September 2009). All necessary informed consent was obtained from the patients or their relatives by the physician in charge. From September 2009, the agreement was waived since the Japanese Ministry of Health, Labor and Welfare judged that the patients’ agreement was not necessary. Patients were registered by the patient registration center, and serial data regarding the AT activity, SIRS score, coagulation markers, and DIC scores were obtained on the day before AT administration (day 0) and on days 2, 4, and 7 after AT administration. DIC was judged to have been resolved when the DIC score had decreased to less than 4 as of day 7. Survival was recorded on day 28. The total bleeding events, including both major and minor bleeding events, were recorded throughout the observation period. Scoring was performed at each hospital.

Either 1,500 IU/day (AT1500) or 3,000 IU/day (AT3000) of AT concentrate (Nihon Pharmaceutical Co. Ltd., Tokyo, Japan) was administered for 3 consecutive days when the patients met the JAAM DIC criteria and had an AT activity level of less than 40%. AT activity was measured at the time of the diagnosis of DIC. The use of AT3000 is approved only for obstetric or surgical DIC patients, and dose adjustment was permitted, depending on the severity. However, since no further details were defined, each doctor decided the dosage on an individual case basis. Standard sepsis care was performed, and platelet concentrate and fresh frozen plasma were used as substitution therapy if necessary, as per the guidelines established by the Japanese Ministry of Health, Labor and Welfare.

### Laboratory measurements

Platelet count, coagulation, and fibrinolytic markers were prospectively assessed. When several sets of data were available on a single day, the least favorable measurement value was used for the analysis. The methods that were used to measure platelet count and coagulation included platelet counting (electric impedance method), prothrombin time (scattered light detection), FDP (latex immunoassay), and D-dimer (latex immunoassay). To measure the AT activity, the plasma anti-Factor Xa activity or the anti-thrombin activity measured in the presence of 0.22 M NaCl, excluding the influence of heparin cofactor II activity, was assessed (chromogenic substrate method, reference intervals: 70% to 120%). All of the measurements were performed by local laboratories.

### Statistical analysis

The paired or unpaired Wilcoxon signed-rank test was applied for two-group comparisons. The proportions were compared by using the chi-square test or the Fisher’s exact test. A Kaplan-Meier curve was calculated, and the survival difference was examined by using a log-rank test. The common method of logistic regression analysis (the enter method) used the outcome (survived, 1; died, 0) as the criterion variable and the AT variable dose (3000 IU/day, 1; 1,500 IU/day, 0), patient sex (male, 1; female, 0), age, body weight, baseline AT activity, baseline DIC score, baseline platelet count, respiratory tract infection (yes, 1; no, 0), digestive tract infection (yes, 1; no, 0), urinary tract infection (yes, 1; no, 0), biliary tract infection (yes, 1; no, 0), other infection (yes, 1; no, 0), unknown infection focus (yes, 1; no, 0), surgery (yes, 1; no, 0), coadministration of recombinant thrombomodulin (yes, 1; no, 0), and coadministration of heparins (yes, 1; no, 0) as explanatory variables. The stepwise method (a forward selection method based on the likelihood ratio) used 28-day survival as the criterion variable and patient sex, age, body weight, baseline AT activity, baseline DIC score, baseline platelet count, respiratory tract infection, digestive tract infection, urinary tract infection, biliary tract infection, other infection, unknown infection focus, surgery and coadministration of recombinant thrombomodulin, coadministration of heparins, and the AT variable dose as explanatory variables. Results were reported as ORs, *P* values, and 95% confidence intervals. For all of the reported results, a *P* value of less than 0.05 was considered to be statistically significant. The above-mentioned analyses were performed by using SPSS 13.0 for MAC OSX (SPSS Inc., Chicago, IL, USA).

## Results

In total, 307 patients were analyzed in the study. Forty-eight patients were treated with AT3000 for 3 days, and 259 patients were treated with AT1500 for 3 days. The mean body weights were 52.3 kg in the AT1500 group and 52.7 kg in the AT3000 group (*P* = 0.863).

Table 1 shows the baseline characteristics of the patients. Respiratory tract infection was the most common underlying disease in the AT1500 group, and digestive tract infection was the most frequent in the AT3000 group. In regard to background differences, digestive tract infection was more common in the AT3000 group (*P* <0.01), and surgery was more common in the AT3000 group (*P* <0.01). In regard to disease severity, the JAAM DIC score, the AT activity, and the SOFA score were identical between the two groups. The baseline platelet count in the AT1500 group was 6.0 × 10^4^/mm^3^ (interquartile range [IQR] 3.15 to 9.1), which was significantly lower than that of the AT3000 group (7.9 × 10^4^/mm^3^, IQR 5.0 to 11.4, *P* <0.05). Heparin was used in 24.7% and 16.7% of the patients in the AT1500 and AT3000 groups, respectively.

**Table 1:** **Baseline patient demographics**

	**AT1500 group n = 259**	**AT3000 group n = 48**	***P*** **value**
Gender			
Male	138 (53.3%)	27 (56.3%)	
Female	121 (46.7%)	21 (43.8%)	
Age in years			
14 or under	1 (0.4%)	0 (0.0%)	
15-64	60 (23.2%)	13 (27.1%)	
65 or over	198 (76.4%)	35 (32.9%)	
Suspected source of infection			
Respiratory tract	85 (32.8%)	11 (22.9%)	
Digestive tract	78 (30.1%)	25 (52.1%)	<0.05
Urinary tract	19 (7.3%)	2 (4.2%)	
Biliary tract	28 (10.8%)	4 (8.3%)	
Others	26 (10.0%)	6 (12.5%)	
Unknown	35 (13.5%)	2 (4.2%)	
Surgery	84 (34.0%)	27 (56.3%)	<0.05
Baseline AT activity			
<30%	88 (34.0%)	19 (39.6%)	
30%	171 (66.0%)	29 (60.4%)	
SD	31.02 ± 7.25	29.57 ± 8.22	
Baseline DIC score			
4.5	143 (55.2%)	26 (54.2%)	
6	48 (18.5%)	10 (20.8%)	
7.8	68 (26.3%)	12 (25.0%)	
SD	5.6 ± 1.3	5.7 ± 1.4	
Baseline SOFA score			
0-6	27 (26.5%)	5 (31.3%)	
7-12	46 (45.1%)	9 (56.3%)	
13-24	29 (28.4%)	2 (12.5%)	
SD	10.1 ± 4.7	7.7 ± 4.3	
Median baseline platelet count, × 10^4^/mm^3^	6.0 (IQR 3.15-9.1)	7.9 (IQR 5.0-11.4)	<0.05
Median baseline FDP, μg/mL	22.1 (IQR 11.5-37.8)	23.7 (IQR 5.0-11.4)	<0.05
Heparin	64 (24.7%)	8 (16.7%)	
Thrombomodulin	15 (5.8%)	3 (6.3%)	

Values are presented as number (percentage) unless otherwise indicated. AT, antithrombin; DIC, disseminated intravascular coagulation; FDP, fibrin/fibrinogen degradation product; IQR, interquartile range; SD, standard deviation; SOFA, Sepsis-Related Organ Failure Assessment.

Among all of the patients, bleeding events were observed in 19 patients (6.88%), and 9 (3.26%) of these were classified as severe. Among the severe bleeding cases, 5 occurred in patients with AT substitution without concomitant unfractionated heparin administration (2.18%), and 4 occurred in patients with unfractionated heparin administration (8.51%) (*P* = 0.049). Among the 9 severe bleeding cases, 7 patients (3.04%) were treated with AT1500 and 2 were treated with AT3000 (4.35%); this difference was not significant (*P* = 0.648) (Table 2).

**Table 2:** **Bleeding events**

**Total**				
	**Any bleeding**		**Major bleeding**	
Without concomitant unfractionated heparin (n = 229)	13	(5.68%)	5	(5.68%)
With concomitant unfractionated heparin (n = 47)	6	(12.77%)	4	(8.51%)
Total (n = 276)	19	(6.88%)	9	(3.26%)
**AT1500 group**				
	**Any bleeding**		**Major bleeding**	
Without concomitant unfractionated heparin (n = 170)	6	(3.53%)	3	(1.76%)
With concomitant unfractionated heparin (n = 60)	10	(16.67%)	4	(6.67%)
Total (n = 230)	16	(6.96%)	7	(3.04%)
**AT3000 group**				
	**Any bleeding**		**Major bleeding**	
Without concomitant unfractionated heparin (n = 38)	3	(7.89%)	2	(5.26%)
With concomitant unfractionated heparin (n = 8)	0	(0.0%)	0	(0.0%)
Total (n = 46)	3	(6.52%)	2	(4.35%)

Values are presented as number (percentage).

**Figure 1:**
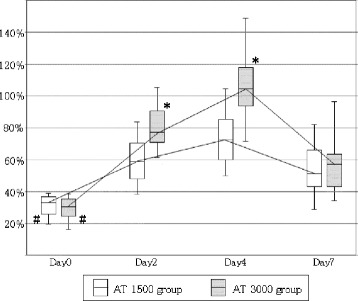
**Changes in antithrombin (AT) activity.** The median baseline AT activity was less than 40% in both groups. AT activities increased to the normal range (>80%) in AT3000 group, but this level was not recovered in the AT1500 group throughout 7 days. AT1500: 1,500 IU/day of AT substitution for 3 days; AT3000: 3,000 IU/day of AT substitution for 3 days. The central horizontal bars, columns, and peripheral horizontal bars indicate the median values, the 25th to 75th percentiles, and the 10th to 90th percentiles, respectively. *P <0.001 versus AT1500 at each day. ^#^ *P* <0.001 versus AT activity of each group at day 2, 4, or 7.

**Figure 2:**
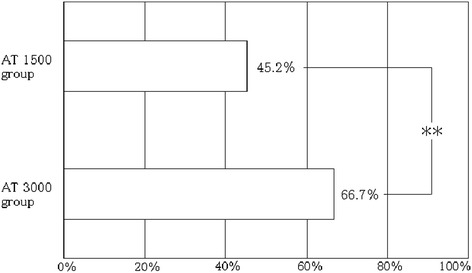
**Comparison of disseminated intravascular coagulation (DIC) resolution rates.** The DIC resolution rates on day 7 were 45.2% in the group with 1,500 IU/day of antithrombin substitution for 3 days (AT1500 group) and 66.7% in the group with 3,000 IU/day of antithrombin substitution for 3 days (AT3000 group). The difference in the DIC resolution rates between these two groups was significant (**P = 0.007, Fisher’s exact test).

**Figure 3:**
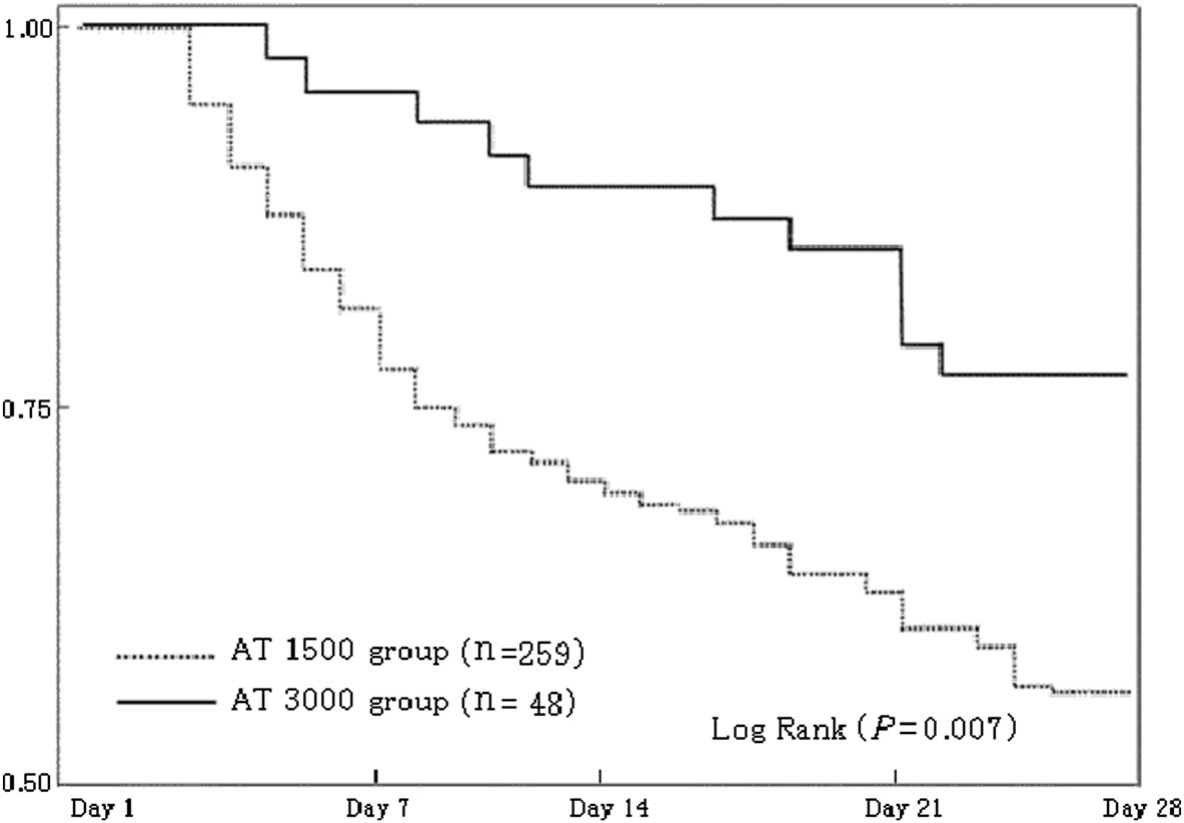
**Comparison of survival.** A Kaplan-Meier curve was calculated to compare the survival between the patients treated with 1,500 IU/day of antithrombin and the patients treated with 3,000 IU/day of antithrombin. The survival started to decrease from day 3 in the AT1500 group and from day 5 in the in the AT3000 group. A significant difference between the groups was recognized on day 28 (*P* = 0.007, log-rank test).

Figure 1 shows the changes in AT activities and the administered AT dose. The AT activities had increased to within the normal range (>80%) in the AT3000 group, but this level was not reached in the AT1500 group throughout the 7 days of observation. As for DIC resolution, AT3000 resulted in a significant reduction in the DIC score, leading to a better recovery from DIC compared with the AT1500 group (66.7% versus 45.2%, *P* = 0.007) (Figure 2). A Kaplan-Meier survival curve was calculated by using data obtained for a maximum of 28 days after the administration of AT. The survival started to decrease from day 3 in the AT1500 group and from day 5 in the AT3000 group, and a significant difference between the groups was recognized (*P* = 0.007) (Figure 3). The AT3000 group exhibited a better survival outcome than the AT1500 group (77.1% versus 56.4%, *P* = 0.010 [Fisher’s exact test], *P* = 0.007 [log-rank test]).

The enter method of logistic regression analysis showed that the supplemented AT dose, patient age, and baseline platelet count were significant factors for survival; that is, the AT dose (OR 2.419, *P* = 0.025), patient age (OR 0.977, *P* = 0.045), and baseline platelet count (OR 1.054, *P* = 0.027) contributed to an improvement in survival. In contrast, neither digestive tract infection nor surgery contributed to the survival (Table 3). A forward selection method based on the likelihood ratio of logistic regression analysis revealed that supplemented AT dose, baseline platelet count and the coadministratrion of recombinant thrombomodulin were significant factors for survival; that is, the AT dose (OR 2.387, *P* = 0.020), baseline platelet count (OR 1.071, *P* = 0.002), and recombinant thrombomodulin (OR 3.853, *P* = 0.038) contributed to improved survival (Table 4).

**Table 3:** **Relationship between outcome (28-day survival) and various factors using the enter method of logistic regression analysis**

	**Odds ratio**	***P*** **value**	**95% confidence interval**
AT dose:			
3,000 versus 1,500 IU	2.414	0.025	1.115-5.226
Gender	0.746	0.296	0.432-1.291
Age	0.977	0.045	0.955-0.999
Body weight	0.998	0.901	0.974-1.023
Baseline AT activity	1.004	0.822	0.969-1.041
Baseline DIC score	0.875	0.198	0.714-1.072
Heparin	0.808	0.499	0.435-1.500
Recombinant thrombomodulin	3.639	0.057	0.960-13.790
Baseline platelet count	1.054	0.027	1.006-1.104
Respiratory tract infection	0.632	0.492	0.170-2.343
Digestive tract infection	1.034	0.960	0.279-3.832
Urinary tract infection	3.019	0.140	0.695-13.115
Biliary tract infection	1.208	0.791	0.300-4.871
Other infection	0.799	0.772	0.175-3.640
Unknown infection focus	0.901	0.891	0.203-3.995
Surgery	0.929	0.818	0.498-1.733

AT, antithrombin; DIC, disseminated intravascular coagulation.

**Table 4:** **Relationship between the outcome (28-day survival) and various factors using the forward selection method based on the likelihood ratio of logistic regression analysis**

	**Odds ratio**	***P*** **value**	**95% confidence interval**
Baseline platelet count	1.071	0.002	1.025-1.119
Recombinant thrombomodulin	3.853	0.039	1.072-13.855
Antithrombin dose			
3,000 versus 1,500 IU	2.387	0.020	1.144 -4.983

## Discussion

The effectiveness of a supplementation dose of AT for septic DIC is controversial. Sawamura and colleagues [20] performed a retrospective analysis in 23 DIC patients and reported that 60 IU/kg per day of AT did not show any advantages for the recovery of platelet counts, coagulation and fibrinolytic markers, and DIC scores, compared with 30 IU/kg per day of AT. In the current survey, AT3000 was found to improve the outcome of sepsis-associated DIC patients significantly, compared with AT1500. The differences between their study and ours were not only the numbers of patients but also the initial AT activity. Since the first survey demonstrated that the efficacy of AT3000 was more prominent in lower AT activity, we focused on the patients with AT activities of less than 40% in the present study.

As for the relationship between the recovered level of AT activity and efficacy, Gando and colleagues [21] retrospectively evaluated the changes in the AT activity after supplementation with a fixed dose of AT (1,500 IU/day or 30 IU/kg per day) and reported that patients who achieved an AT activity level of more than 60% had a better outcome. In our survey, the AT activity reached over 80% in the AT3000 group but was around 60% in the AT1500 group. However, whether the recovered AT activity was a result of the supplementation or the cause of the improvement in survival is not clear. A target activity-oriented AT supplementation study should be performed to address this issue.

The main concern regarding the use of AT is the possible increase in the risk of bleeding. When high-dose AT, in particular, was administered with either unfractionated or low-molecular-weight heparin, the number of bleeding events increased significantly (23.8% for AT with heparin versus 13.5 for placebo; *P* <0.001) in KyberSept [5]. In contrast, heparin use did not affect the incidence of bleeding events in our former survey. A total of 193 out of 729 patients (26.5%) received heparin, and neither the incidences of bleeding events (6.55% with heparin versus 6.51% without heparin) nor the increase of serious bleeding events (1.79% with heparin versus 1.68% without heparin) differed between patients treated with and those treated without heparin. In the present study, the number of bleeding events was more than two times higher in patients with concomitant heparin use, compared with those receiving AT alone. We suspect that the severity of sepsis and the degree of coagulation disorder might have influenced this difference, but further examination is needed to clarify this issue. In regard to the bleeding risk without heparin treatment, the rate was not significantly higher than that observed in the former survey regardless of the increase in coagulation disorder. There was no intracranial hemorrhage and deadly bleeding in this study; however, it should be kept in mind that the rate of severe bleeding was doubled in the present study.

Interestingly, the coadministration of recombinant thrombomodulin was revealed to be another significant factor for survival by the forward selection method of logistic regression analysis. Because both AT and thrombomodulin activities are known to decrease significantly during severe sepsis and their mechanisms of action are independent, a combination therapy could potentially be beneficial. On the basis of this hypothesis, we examined the combination effects in the lipopolysaccharide-challenged animal model and reported that the combination therapy attenuates organ damage, causes histologic changes, and leads to an improvement in survival [22,23]. However, the efficacy has not been proven in the clinical field yet. Furthermore, since the *P* value did not reach significant in the enter method, this issue should be discussed in the next study.

Finally, the present study had several limitations. First, the superiority of the AT3000 regimen might be related to the surgery rather than the AT volume. As was shown by the stepwise logistic regression analysis, surgery was a significant factor in the use of AT3000, and this result can be explained by the Japanese health-care system. Since DIC patients who require surgical treatment and obstetric DIC patients have lower AT activity levels, the use of AT3000 is approved for these patients. To answer the question of whether the surgery itself, rather than the AT3000 treatment, might have improved the outcome, it should be noted that since the enter method of logistic regression analysis revealed that surgery was not a significant explanatory variable for the outcome, the AT3000 regimen might be responsible for the improvement in survival. A second limitation is that the dose allocations were not randomly performed. Although the baseline SOFA score was not different between the groups, the ratio of very severe cases (SOFA score >12) was higher in the AT1500 group than in the AT3000 group (28.4% versus 12.5%), and this might have affected the results. Third, a placebo control was not included in the study. Fourth, the patient number in the AT3000 group was relatively small. In addition, we cannot eliminate the influence of the higher baseline platelet count in the AT3000 group. Therefore, we think that the survey should be continued.

## Conclusions

The supplementation of AT at a dose of 3,000 IU/day (compared with 1,500 IU/day) for 3 days resulted in the effective modulation of sepsis-associated DIC resolution and an improvement in the 28-day survival outcome without increasing the risk of bleeding in DIC patients with a baseline AT activity level of less than 40%. Although the results of the present study may provide a rationale for the use of a supplemental dose of AT, a large-scale RCT addressing the efficacy and bleeding risk of AT for DIC in patients with severe sepsis is required.

## Key messages

- AT supplementation at 3,000 IU/day improved the outcome of sepsis-associated DIC in patients with a baseline AT activity level of less than 40%, compared with 1,500 IU/day of AT.
- AT supplementation at 3,000 IU/day does not increase the bleeding risk, compared with AT supplementation at 1,500 IU/day.
- The concomitant use of heparin may increase the bleeding risk.

## Abbreviations

AT: antithrombin
DIC: disseminated intravascular coagulation
FDP: fibrin/fibrinogen degradation product
IQR: interquartile range
JAAM: Japanese Association for Acute Medicine
OR: odds ratio
RCT: randomized controlled trial
SIRS: systemic inflammatory response syndrome
SOFA: Sepsis-Related Organ Failure Assessment
